# Recurrent Testicular Torsion of a Fixed Testis

**DOI:** 10.1155/2019/8735842

**Published:** 2019-07-15

**Authors:** Ibrahim Alnadhari, Ausama Abdulmuhsin, Omar Ali, Ahmad Shamsodini, Morshed Salah, Osama Abdeljaleel

**Affiliations:** Division of Urology, Department of Surgery, Al Wakra Hospital, Hamad Medical Corporation, Doha, Qatar

## Abstract

Recurrent testicular torsion after previous orchiopexy is rare and needs high index of suspension to avoid misdiagnosis and delayed management. This case showed that this diagnosis can occur even when the testis is still fixed to the scrotal wall. A 31-year-old male who had previous testicular fixation for testicular torsion with a single stitch to the lower pole before 6 years presented with recurrent testicular torsion and missed diagnosis. This case confirm that recurrent testicular torsion after previous fixation should be included in the differential diagnosis of acute scrotum and emphasis on the testicular fixation with nonabsorbable suture in at least two points to prevent recurrent torsion.

## 1. Introduction

Testicular torsion is considered one of the important causes of acute scrotum condition. It is most common just after birth and during puberty. It occurs in about 1 in 4,000 to 1 per 25,000 males per year before 18 years of age [1]. Although its overall incidence is not high, it remains the most common cause of testicular loss in these age groups. Yet, torsion may occasionally occur in men of 40-50 years old.

Testicular torsion is mainly diagnosed by history and physical examination then immediate surgical exploration is indicated but scrotal ultrasonography can be useful diagnostic tool if it is available and there is doubt in diagnosis [2]. Late presentation to hospital and delay due to hospital transfer were the major risk factors for delayed management and orchiectomy in such patients [3]. Recurrent testicular torsion after previous orchiopexy is rare and needs high index of suspension to avoid misdiagnosis and delayed management. Hereby we present a case of missed recurrent testicular torsion after previous orchiopexy.

## 2. Case Report

**Figure 1:**
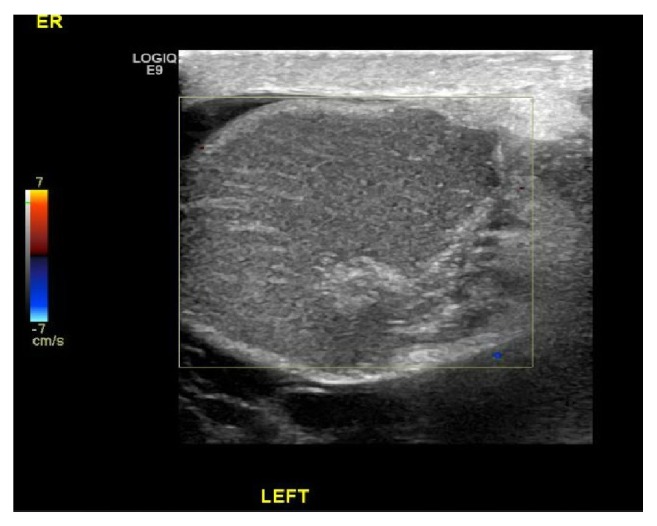
Scrotal ultrasonography showed absent testis blood flow.

A 31-year-old male patient presented to the emergency complaining of left testicular pain for 5-day duration since the onset of pain, continuous pain associated with swelling, gradually increased. He had previously undergone bilateral orchidopexy before 6 years for left testicular torsion. Patient visited general practitioner on the same day of onset of pain and was diagnosed as epididymitis and given antibiotics with analgesics and discharged. On examination the entire left hemiscrotum was swollen and tender and appeared as a confluent mass without identifiable landmarks. Scrotal ultrasonography showed hypoechoic left testis without intrinsic vascularity (Figure 1) and moderate to gross left hydrocoele is noted with internal echoes and septation.

**Figure 2:**
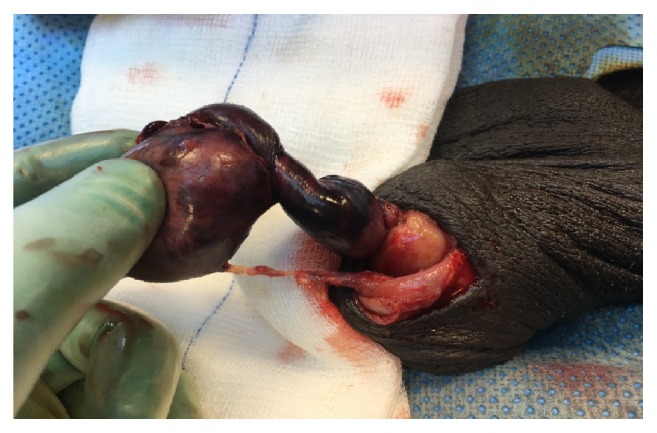
Black, torsed left testis attached with old stitch from lower pole to the side wall of scrotum.

Urgent surgical exploration of left side revealed intact tunica vaginalis with moderate hydrocele, left testis delivered which was black, torsed, and attached with old stitch from lower pole to the side wall of scrotum (Figure 2). Old stitch was removed and the testis was detorsed but it was gangrenous so orchiectomy done.

**Figure 3:**
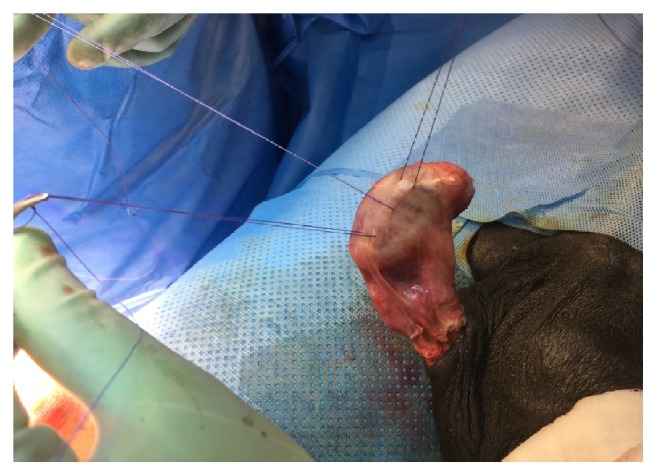
Fixation of the right testis with three stitches.

The right testis was explored and it was attached with single stitch to the lower pole; old stitch was removed and fixation with three stitches 3/0 vicryl to the scrotal wall was done (Figure 3). Patient was discharged on the first postoperative day and he had uneventful postoperative course. He was given clear instructions to avoid trauma to his single testis and to report to the emergency in case of testicular pain.

## 3. Discussion

Early diagnosis and management of testicular torsion is important to save the testis. Correction within 4-6 hours gives good testicular preservation but if it is done after 12 hours there is high risk of testicular atrophy [2].

The diagnosis of testicular torsion after previously fixed testicles is rare but it should be included in the differential diagnosis of acute scrotum.

There have been reported cases of recurrent testicular torsion after the previous [4–14]. Some of them were diagnosed early and second time orchiopexy was done [4–7, 9, 12–14] and others were delayed in the diagnosis and underwent orchiectomy [8, 10, 11]. In most of the cases, they used absorbable suture and during exploration the testicle was found freely hanging and lost its attachment to the scrotal wall or tunica vaginalis.

As in our case, only two reported cases with torsion occurred while the testis is attached to the scrotum through the stitch in the lower pole [13, 14]. Single lower pole stitch makes the testis hanged “like a hammock” which increase its risk of rotation and torsion.

Fixation of the detorsed testis and the contralateral testis using nonabsorbable suture is recommended to prevent metachronous or recurrent torsion [15]. Fixation of the testis in at least two points theoretically prevents torsion.

Testicular torsion leading to orchidectomy is a major catastrophe for the patient and previous history of orchiopexy does not exclude the diagnosis.

## 4. Conclusion

Recurrent testicular torsion after previous orchiopexy is rare but should be included in the differential diagnosis of acute scrotum. Testicular fixation with nonabsorbable suture and in at least two points can help to prevent recurrent torsion.
